# Bidirectional analysis of the association between migraine and post-traumatic stress disorder in Nurses’ Health Study II

**DOI:** 10.1017/S2045796024000799

**Published:** 2024-12-11

**Authors:** H. M. Crowe, L. Sampson, A. C. Purdue-Smithe, K. M. Rexrode, K. C. Koenen, J. W. Rich-Edwards

**Affiliations:** 1 Division of Women’s Health, Department of Medicine, Brigham and Women’s Hospital, and Harvard Medical School, Boston, MA, USA; 2 Department of Epidemiology, Harvard T.H. Chan School of Public Health, Boston, MA, USA; 3 Program in Public Health, Department of Family, Population, & Preventive Medicine, Stony Brook, NY, USA

**Keywords:** epidemiology, population survey, PTSD, risk factors, trauma

## Abstract

**Aims:**

Migraine and post-traumatic stress disorder (PTSD) are both twice as common in women as men. Cross-sectional studies have shown associations between migraine and several psychiatric conditions, including PTSD. PTSD is disproportionally common among patients in headache clinics, and individuals with migraine and PTSD report greater disability from migraines and more frequent medication use. To further clarify the nature of the relationship between PTSD and migraine, we conducted bidirectional analyses of the association between (1) migraine and incident PTSD and (2) PTSD and incident migraine.

**Methods:**

We used longitudinal data from 1989–2020 among the 33,327 Nurses’ Health Study II respondents to the 2018 stress questionnaire. We used log-binomial models to estimate the relative risk of developing PTSD among women with migraine and the relative risk of developing migraine among individuals with PTSD, trauma-exposed individuals without PTSD, and individuals unexposed to trauma, adjusting for race, education, marital status, high blood pressure, high cholesterol, alcohol intake, smoking, and body mass index.

**Results:**

Overall, 48% of respondents reported ever experiencing migraine, 82% reported experiencing trauma and 9% met the Diagnostic and Statistical Manual of Mental Disorders-5 criteria for PTSD. Of those reporting migraine and trauma, 67% reported trauma before migraine onset, 2% reported trauma and migraine onset in the same year and 31% reported trauma after migraine onset. We found that migraine was associated with incident PTSD (adjusted relative risk [RR]: 1.26, 95% confidence interval [CI]: 1.14–1.39). PTSD, but not trauma without PTSD, was associated with incident migraine (adjusted RR: 1.20, 95% CI: 1.14–1.27). Findings were consistently stronger in both directions among those experiencing migraine with aura.

**Conclusions:**

Our study provides further evidence that migraine and PTSD are strongly comorbid and found associations of similar magnitude between migraine and incident PTSD and PTSD and incident migraine.

## Introduction

Migraine and post-traumatic stress disorder (PTSD) are complex, debilitating brain disorders. Migraine is a neurological disorder characterized by unilateral, long-lasting headache pain, classically aggravated by routine physical activity, photophobia/phonophobia, and often accompanied by nausea and/or vomiting. (Olesen, 2024) PTSD is a mental health condition which 2011can arise following exposure to a traumatic event and is characterized by reexperiencing the traumatic event through flashbacks or nightmares, irritability, poor concentration, hypervigilance, difficulty sleeping and emotional withdrawal (Zarei *et al.*, 2016).

Migraine and PTSD are heterogenous in presentation, compromise quality of life, and are two times more common among women than men. (Friedman *et al.*, 2017; Peterlin *et al.,* 2011) Approximately 1 in 4 women in the United States will experience migraine and 1 in 10 will experience PTSD in their lifetime. (Peterlin *et al.*, 2011; Rao *et al.*, 2015) Migraine and PTSD have shared risk factors, including such as childhood maltreatment, exposure to natural disasters, and chronic stress. Migraine and PTSD may also share underlying pathophysiological pathways including genetic factors, serotonergic dysfunction, disruption of ovarian hormones, or central nervous system sensitization. (Dresler *et al.*, 2019; Peterlin *et al.*, 2011; Smitherman and Kolivas, 2013) Although the exact mechanism is unknown, individuals who experience migraine are known to have abnormal stress responses in the central nervous system and the hypothalamic pituitary–adrenal axis. Characteristics of these responses, such as lower heart rate variability and persistently elevated cortisol have also been found in individuals with PTSD, suggesting that people who experience migraine may be more biologically prone to developing PTSD when exposed to trauma. (Peterlin *et al.*, 2011; Sauro and Becker, 2009) This may be especially true for individuals who experience migraine with aura, which has been associated with a greater prevalence of psychiatric comorbidity than migraine without aura (Antonaci *et al.*, 2011).

Previous research has shown an association between migraine and several psychiatric conditions, including PTSD. (Minen *et al.*, 2016) Studies of headache clinic patients show increased prevalence of PTSD compared to the general population, and greater headache-related disability among individuals with comorbid migraine and PTSD compared to those with migraine without PTSD. (Lipton *et al.*, 2020; Rammohan *et al.*, 2019; Zarei *et al.*, 2016) Longitudinal studies have established bidirectional associations between migraine and depression and anxiety. In particular, migraine has been consistently associated with an increased risk of developing depression. (Breslau and Davis, 1993; Breslau *et al.*, 2000; Modgill *et al.*, 2012) However, epidemiologic data on the longitudinal association between migraine and PTSD are limited to a single study of incident migraine among 5,644 PTSD patients and 22,576 age- and sex-matched controls in Taiwan. Patients with PTSD were almost four times as likely to develop migraine (hazard ratio [HR] = 3.83, 95% CI:2.28–5.20) as individuals without PTSD. Researchers also found a dose-response relationship between PTSD severity (defined as number of annual visits to a PTSD clinic) and rate of migraine (Huang *et al.*, 2019).

While PTSD and migraine were assessed simultaneously in the National Comorbidity Study Replication (NCS-R), approximately 70% of the 120 participants reporting headache and PTSD reported that they experienced PTSD onset prior to the development of severe or frequent headaches. (Rao *et al.*, 2015) While both studies support a clear association between these conditions, neither study was able to establish clear temporality of PTSD and migraine onset, as the NCS-R was cross-sectional and Huang et al. defined PTSD onset as date of enrollment in the study and only considered those with a formal diagnosis of migraine as already having the outcome at baseline.

The purpose of this study was to analyze the longitudinal association between migraine and incident PTSD as well as PTSD and incident migraine among U.S. current or former nurses from 1989 to 2020. This novel bidirectional analysis thus sought to quantify the direction and magnitude of these associations. Based on previous research, we hypothesized that women with migraine, particularly those with migraine with aura, would have an increased risk of developing PTSD after exposure to trauma. We also hypothesized that PTSD would be associated with an increased risk of developing migraine, particularly migraine with aura.

## Methods

### Study population

We analyzed data from the Nurses’ Health Study II (NHS II), an ongoing prospective cohort study that began in 1989, which comprised 116,429 female registered nurses aged 25–42 at baseline. NHS II participants complete follow-up questionnaires every 2 years. We linked data from the main NHS II questionnaire to a 2018 questionnaire on trauma experiences of a subset NHS II participants. Sub-study participation has been detailed elsewhere. (Sampson *et al.*, 2022) Briefly, we contacted 51,486 active NHS II participants with a known email address in August 2018 and invited them to complete a web-based PTSD questionnaire. Between August 2018 and January 2020, 33,845 participants (65.7%) responded. We excluded 229 participants who did not complete the required trauma section and 289 participants who did not complete the PTSD symptom section, for a final analytic sample size of 33,327 participants. There were no substantial differences in demographic factors between responders and non-responders. The study protocol was approved by the Institutional Review Board of the Brigham and Women’s Hospital and the Harvard T.H. Chan School of Public Health and return of the questionnaires was considered implied consent.

### Assessment of migraine

We ascertained migraine through participant responses on the main NHS II questionnaires. The questionnaires administered in 1989, 1993 and 1995 asked participants if they had ever been diagnosed with migraine by a healthcare provider. In 2007, participants indicated if they had ever experienced a migraine headache and, if so, if they experienced an aura. The 2009 and 2013 questionnaires assessed if the participants had a migraine in the past 2 years, and, if so, if they experienced an aura. Participants who indicated migraine diagnosis or experiencing a migraine on any questionnaire were considered to have migraine and participants who ever indicated having experienced a migraine with aura were considered to have migraine with aura.

### Assessment of trauma exposure and PTSD

We assessed PTSD by participant responses on the 2018 stress questionnaire. First, we assessed lifetime traumatic life events using a 16-item modified version of the Brief Trauma Questionnaire. (Sampson *et al.*, 2022; Schnurr 1999) Respondents who reported at least one trauma were then asked to specify which event they considered their worst and their age when it occurred. Trauma-exposed participants were also asked to indicate which type(s) of trauma they experienced, which were then combined into the following categories: sexual/interpersonal, accident/disaster, sudden death of a loved one, illness/injury, nursing-related or ‘other’. Probable PTSD was assessed using a modified version of the self-report PTSD Checklist for the Diagnostic and Statistical Manual of Mental Disorders (DSM), Version 5 (PCL-5). (Blevins *et al.*, 2015) Participants first indicated whether they had ever experienced each symptom in their lifetime (following their worst trauma), and then indicated the degree to which they experienced each symptom in the past month. We determined probable lifetime PTSD using the DSM-5 criterion definition. In separate models PTSD was operationalized both as a binary (yes/no) or categorical (PTSD, trauma without PTSD, no trauma) variable. Participants also indicated whether they had ever been treated for PTSD.

### Assessment of condition onset

**Figure 1.**
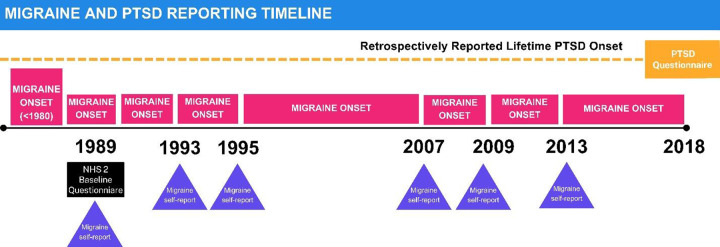
Timeline of migraine and PTSD reporting.

As this was a bidirectional analysis, determining the temporality of both migraine and PTSD onset was critical. Based on specific questionnaire wording we were able to categorize migraine onset as: before 1980, 1980–1984, 1985–1989, 1990–1991, 1992–1993, 1994–1995, 1996–2007, 2008–2009 and 2010–2013 (Fig. 1). Migraine onset was set to the latest possible date of first occurrence. For example, a participant reporting migraine onset between 1994 and 1995 would be considered to have migraine onset on December 31^st^, 1995.

Date of PTSD onset was not directly queried in the 2018 questionnaire and was therefore defined as the date of worst event (year of birth + age at worst event). Data from a 2008 questionnaire in the same cohort revealed that the majority (70%) of participants reported that PTSD symptom onset occurred within one year of their worst traumatic event. We compared the date of PTSD and migraine onset (if applicable), and categorized participants as migraine onset before PTSD, PTSD onset before migraine, or undetermined (PTSD and migraine onset occurred in the same year).

### Assessment of covariates

We collected covariate data, including demographic, medical and lifestyle factors, from the main NHS II questionnaires. Potential confounders ascertained on the baseline questionnaire included race (dichotomized as non-Hispanic white and other race due to small numbers of non-white participants), education level (associates, masters, doctorate), marital status (married, divorced, separated, widowed, partnership, single). We also collected data on ever-diagnosis of high blood pressure (yes/no), and/or high cholesterol (yes/no), alcohol intake (g/day), selective serotonin reuptake inhibitor (SSRI) use (yes/no), smoking status (yes/no) and Body Mass Index (BMI; kg/m^2^, continuous) from the 2017 questionnaire. We examined concurrent positive screening for clinical depression (Center for Epidemiologic Studies Depression Scale score of ≥16) on the 2018 stress questionnaire as a correlate of interest due to high symptom overlap and cooccurrence of depression and PTSD. We used the missing-indicator method (Groenwold *et al.*, 2012) for missing confounder data, as missingness was low (≤3%) for all variables (Table 1).

**Table 1.** Characteristics of the NHS2 participants completing the NHS2 2018 stress questionnaire by migraine status

Characteristic^a^	Incident PTSD cohort (*N* = 23,892)		Incident migraine cohort (28,815)		Entire study sample (33,327)	
**Migraine status**	No reported migraine (*N* = 17,221, 72%)	Ever reported migraine^b^ (*N* = 6,671, 27%)	No reported migraine (*N* = 17,221, 60%)	Ever reported migraine (*N* = 11,594, 40%)	No reported migraine (*N* = 17,221, 52%)	Ever reported migraine^b^ (*N* = 16,106, 48%)
Age in 2018, mean	64.8 (4.6)	64.4 (4.6)	64.8 (4.6)	64.5 (4.6)	64.8 (4.6)	64.5 (4.6)
Non-Hispanic white, %	96	96	96	96	96	96
**Education**						
Associates, %	24	26	24	24	24	25
Bachelors, %	40	40	40	39	40	40
Masters, %	28	26	28	28	28	28
Doctorate, %	5	4	5	5	5	5
**Marital status**						
Married, %	74	74	74	75	74	74
Divorced, %	11	11	11	12	11	12
Separated, %	<1	1	<1	<1	<1	1
Widowed, %	6	8	6	5	6	6
Partnership, %	2	1	2	1	2	1
Single, %	6	5	6	5	6	5
**Medical/Lifestyle factors**						
Ever diagnosis of high blood pressure, (2017)%	25	28	28	30	25	28
Ever diagnosis of high cholesterol, (2017)%	28	30	28	30	28	30
Screened positive for clinical depression (CES-D,^c^ 2018)%	18	26	19	24	18	25
SSRI^c^ use (2017)	12	15	12	15	12	16
Alcohol g/day (2017)	2.5 (5.5)	1.9 (4.4)	2.5 (5.5)	2.1 (4.9)	2.5 (5.5)	2.1 (4.8)
Current smoker, % (2017)	3	3	3	3	3	3
BMI^c^, kg/m^2^ (2017)	27.4 (6.2)	27.7 (6.4)	27.4 (6.2)	27.7 (6.3)	27.4 (6.2)	27.8 (6.3)

a Self-reported physician-diagnosed migraine (pre-2007), self-reported migraine headache (2007–2020).

b Missingness for education level depression was 3%, missingness for marital status, and cigarette smoking was <1%. All other variables had no missing values.

c CES-D: Center for Epidemiological Studies Depression Scale. SSRI: selective serotonin reuptake inhibitor. BMI: body mass index.

### Statistical analysis

We conducted several descriptive analyses to characterize migraine and trauma/PTSD in our population. We first used bivariate analysis to compare the distribution of potential covariates among individuals ever reporting migraine and never reporting migraine. Among individuals with probable PTSD, we examined the distribution of migraine by reported trauma type, to determine if the women with migraine were more likely to experience certain types of traumas and used a chi-square test to test for a cross-sectional association between migraine status and PTSD treatment. We then conducted a cross-sectional analysis to calculate the prevalence odds ratio of migraine and PTSD among participants. We repeated this analysis, restricting to individuals who reported exposure to trauma.

**Figure 2.**
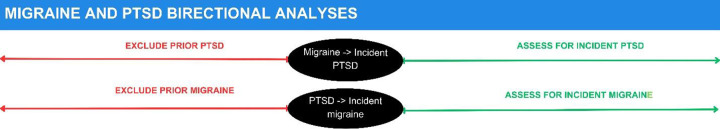
Bidirectional assessment model.

For the analyses of migraine history predicting incident PTSD, we excluded individuals who reported no trauma and those who reported trauma prior to or in the same year as migraine onset to compare incident PTSD among women with migraine, compared with women without migraine (Fig. 2). We used multivariable log-binomial models to estimate crude and adjusted risk ratios for the association between migraine (1979–2013) and incident PTSD (through 2020). In final models we adjusted for race, education, marital status, history of high blood pressure/high cholesterol, alcohol intake, smoking status and BMI. Due to unclear temporality between migraine onset and covariate data, we also fit models which were only adjusted for likely time-invariant covariates (race, education, marital status), and effect estimates were unchanged. We fit similar models with migraine as a three-level exposure: migraine with aura, migraine without aura and no migraine, to assess the association across different migraine phenotypes.

For the PTSD and incident migraine analysis, we excluded individuals who reported migraine onset prior to or in the same year as their worst trauma (Fig. 2). We operationalized PTSD as a three-level categorical variable (no trauma, trauma without PTSD, PTSD) for this analysis and used log-binomial models to estimate the risk of developing incident migraine among those with PTSD and those with trauma exposure who did not screen positive for PTSD, compared to those unexposed to trauma. We ran two adjusted models as described above. To assess the association between PTSD and incident migraine with aura we excluded women without migraine and compared the association between trauma/PTSD and the outcome of migraine with aura (case) versus migraine without aura (non-case).

### Sensitivity analyses: 2008 questionnaire

To further isolate the prospective association between PTSD and incident migraine, we analyzed data from a similar questionnaire completed by 54,224 NHS II participants in 2008. Details of this questionnaire have been described in detail elsewhere. (Sumner *et al.*, 2015) Briefly, participants were asked if they had experienced trauma using the Brief Trauma Questionnaire. Trauma-exposed participants completed a short screening which asked participants to report the presence or absence of seven PTSD symptoms in their lifetime, following their worst traumatic event. Participants were then categorized into the following categories: (1) no trauma exposure, (2) trauma-exposed and endorsed no PTSD symptoms, (3) trauma-exposed and endorsed 1–3 symptoms, (4) trauma-exposed and endorsed 4–5 symptoms, and (5) trauma-exposed and endorsed 6–7 symptoms. We excluded individuals who reported migraine before 2008 and fit log-binomial models to assess the association between trauma exposure and PTSD symptoms and risk of incident migraine, adjusting for covariates measured in 2007.

## Results

### Descriptive results

Participants completing the 2018 stress questionnaire closely resembled the overall NHS II cohort (Supplemental Table S1). Participants were 64 years old on average, and mostly non-Hispanic white (96%). A third of participants had a graduate degree or higher, 74% were married, and very few were current smokers (3%). Approximately one-fifth of participants screened positive for depression on the 2018 stress questionnaire.

**Figure 3.**
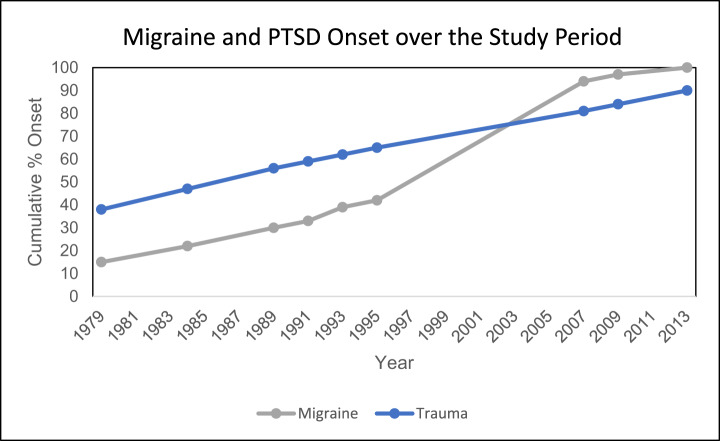
Temporality of migraine and PTSD reporting.

**Figure 4.**
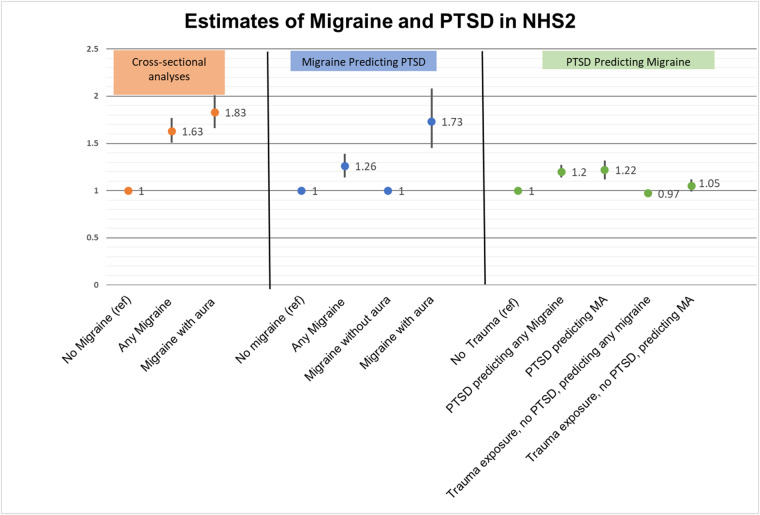
Summary of estimates of the association between migraine and PTSD.

Almost half (48%) of the 33,327 NHS II participants who completed the 2018 stress questionnaire reported ever being diagnosed with or experiencing migraine between 1989 and 2013. Overall, women with and without migraine reported very similar demographic, medical and lifestyle characteristics (Table 1). Women with migraine were more likely to report SSRI use on the 2017 questionnaire (16% vs. 12%) and to screen positive for clinical depression on the 2018 stress questionnaire (25% vs. 18%) than those not reporting migraine. Overall, 82% of participants completing the 2018 stress questionnaire reported having experienced at least one traumatic event, and 9% reported probable PTSD. The most common type of trauma was sudden death of a loved one, followed by sexual/interpersonal trauma. There were no meaningful differences in migraine prevalence by trauma type, considering all trauma reported those with and without migraine (Supplemental Table S2). Of the 13,693 individuals reporting migraine and trauma, 9,181 (67%) reported experiencing trauma prior to migraine onset, 254 reported trauma (2%) and migraine onset in the same year, and 4,258 (31%) reported migraine onset before trauma (Fig. 3). Among individuals with PTSD, individuals with migraine were slightly more likely to report ever receiving PTSD treatment than those without migraine (69% vs. 62%). Cross-sectional and bidirectional results are summarized below and in Fig. 4.

The graph above shows the cumulative proportion of migraine and PTSD reported, among individuals reporting both conditions. Circles represent years in which migraine was assessed on the NHS II questionnaire. On the 1989 NHS II baseline questionnaire, 30% of individuals reported migraine occurring in 1989 or earlier and 56% of individuals had already experienced their worst traumatic event, as retrospectively reported in 2018.

### Cross-sectional results

In cross-sectional analysis, women with migraine were more likely to report lifetime PTSD than women without migraine (adjusted prevalence odds ratio [POR] = 1.63, 95% CI: 1.51–1.77) (Fig. 4). Individuals reporting migraine with aura had increased odds of also reporting lifetime PTSD (RR = 1.83, 95% CI 1.66%–2.01%) compared with those without migraine. These estimates were slightly attenuated when we restricted the analysis to individuals who reported trauma (adjusted POR = 1.53, 95% CI 1.41–1.66 for migraine overall and adjusted POR = 1.68, 95% CI 1.53–1.85 for migraine with aura). Fig. 3

### Migraine and incident PTSD

Excluding individuals who reported trauma before migraine onset, and adjusting for confounders, migraine overall was associated with a moderately increased risk of developing PTSD (RR = 1.26 95% CI: 1.14–1.39) among trauma exposed individuals (Table 2). Individuals reporting migraine with aura had 1.74 times the risk of developing PTSD as individuals without migraine (95% CI: 1.45–2.08) (Table 3).

**Table 2.** Risk of incident PTSD by migraine status among trauma-exposed individuals^a^

	Developed PTSD *N*(%)	Did not develop PTSD *N*(%)	Crude RR (95% CI)	Adj. RR^b^ (95% CI)
Migraine	577 (8.7%)	6,093 (91.4%)	1.27 (1.15–1.40)	1.26 (1.14–1.39)
No migraine	1,174 (6.8%)	16,048 (93.2%)	Ref	Ref

a Excludes individuals who experienced trauma prior to migraine onset.

b Adjusted for race, education, marital status, high blood pressure, high cholesterol, alcohol intake, smoking, BMI.

**Table 3.** Risk of incident PTSD by migraine status among trauma-exposed individuals^a^

	Developed PTSD *N*(%)	Did not develop PTSD *N*(%)	Crude RR (95% CI)	Adj. RR^b^ (95% CI)
Migraine with aura	187 (9.3%)	1,824 (90.7%)	1.78 (1.50–2.11)	1.74 (1.45–2.08)
Migraine without aura	164 (6.4%)	2,418 (93.7%)	1.22 (1.01–1.46)	1.21 (1.00–1.46)
No migraine	342 (5.2%)	6,200 (94.8%)	Ref	Ref

a Excludes individuals who experienced trauma prior to migraine with aura assessment.

b Adjusted for race, education, marital status, high blood pressure, high cholesterol, alcohol intake, smoking, BMI.

### PTSD and incident migraine

Excluding individuals who experienced migraine onset before trauma, PTSD was associated with a 20% increase in the risk of incident migraine (95% CI: 14%–27%) compared to those who reported no trauma exposure (Table 4). Those who were exposed to trauma but did not develop PTSD did not have an increased risk of developing migraine (RR = 0.97, 95% CI:0.94–1.01). Among those with new onset migraine after 2007 (*n* = 8,301, when aura was first measured), those with PTSD experienced 1.22 times (95% CI: 1.12–1.32) the risk of developing migraine with aura. Trauma exposure in the absence of PTSD was not appreciably associated with an increased risk of developing migraine with aura (RR = 1.05, 95% CI:0.99–1.12), as compared to individuals who did not experience trauma (Table 5).

**Table 4.** Risk of incident migraine by PTSD status^a^

PTSD	Migraine *N*(%)	No migraine *N*(%)	Crude RR (95% CI)	Adj. RR^b^ RR (95% CI)
Yes	1,105 (48.5%)	1,174 (51.5%)	1.20 (1.13–1.26)	1.20 (1.14–1.27)
No, trauma exposed	8,076 (39.2%)	12,513 (60.8%)	0.97 (0.93–1.00)	0.97 (0.94–1.01)
No, no trauma	2,412 (40.1%)	3,535 (59.4%)	Ref	Ref

*Excludes individuals who experienced migraine onset before trauma.

b Adjusted for race, education, marital status, high blood pressure, high cholesterol, alcohol intake, smoking, BMI.

**Table 5.** Risk of incident migraine with and without aura by PTSD status^a^

PTSD	Migraine with aura	Migraine without aura	Crude RR	Adj. RR^b^
Yes	324 (40.5%)	476 (59.5%)	1.26 (1.13–1.42)	1.22 (1.12–1.32)
No, trauma exposed	2,171 (35.2%)	3,998 (64.8%)	1.10 (1.01–1.19)	1.05 (0.99–1.12)
No, no trauma	427 (32.1%)	904 (67.9%)	Ref	Ref

a Among those with migraine, excluding migraine before 2007.

b Adjusted for race, education, marital status, high blood pressure, high cholesterol, alcohol intake, smoking, BMI.

### Sensitivity analyses

In total, 5% (*n* = 1,361) of participants without a history of migraine in 2008 reported new onset migraine in 2009 or 2013. Rates of trauma exposure were similar between those who reported new onset migraine and those who did not develop migraine (80% vs. 78%). Participants who reported trauma without endorsing PTSD symptoms had a slightly elevated risk of developing migraine as compared to those who did not experience trauma (RR = 1.14, 95% CI: 0.95–1.38) (Supplemental Table S2). Participants who reported 1–5 PTSD symptoms had a similar risk of developing migraine as participants who did not experience trauma, however participants reporting 6–7 symptoms had 1.77 times (95% CI: 1.32–2.37) the risk of developing migraine as participants who were not trauma exposed. Overall, 25% (*n* = 335) of participants who reported new onset migraine experienced migraine with aura. We did not find an association between trauma exposure in the absence of PTSD symptoms and risk of developing migraine with aura (RR = 1.06, 95% CI:0.77–1.53) (Supplemental Table S3). We found varying degrees of risk of incident migraine with aura for individuals reporting 1–3 PTSD symptoms (RR = 1.36, 95% CI: 0.89–2.07), 4–5 PTSD symptoms (RR = 1.66, 95% CI: 1.06–2.58), or 6–7 PTSD symptoms (RR = 1.28, 95% CI: 0.77–2.14), with no clear dose-response pattern.

## Discussion

We found associations of similar magnitude when examining the cross-sectional association between migraine and PTSD, the longitudinal association between migraine and incident PTSD, and the longitudinal association between PTSD and incident migraine (Fig. 4). While this longitudinal dataset allowed us to establish that migraine history predicts incident PTSD and that PTSD history predicts incident migraine, the temporality of the associations gives us only limited insight into whether these conditions cause each other: migraine may increase PTSD risk, PTSD may increase migraine risk, and/or their association may reflect shared common causes.

While this is the first study to longitudinally assess the association between migraine and incident PTSD, investigators have posited that individuals with migraine may be biologically predisposed to developing PTSD if they are exposed to traumatic events. Individuals with migraine have increased baseline cortisol levels, and lower reactive levels of cortisol, which is consistent with patterns known to be associated with the development of PTSD following a traumatic event (Minen *et al.*, 2016).

The present study is consistent with previous research in that PTSD symptoms preceded migraine symptoms in most participants reporting both conditions (67% in our study and 69% in previous research), potentially due to trauma exposure occurring early in life, prior to the onset of migraines. (Peterlin *et al.*, 2011) Previous longitudinal research on the development of migraine among PTSD patients found that individuals with PTSD had 3.83 (95% CI: 2.82–5.20) times the risk of developing migraine later in life than age and sex-matched controls. (Huang *et al.*, 2019) While our findings are directionally consistent with this study, we found a weaker association between PTSD and incident migraine. This may partially be explained by our study including only females, as previous research found that while migraine and PTSD are more common in women than men, the association between episodic migraine and lifetime PTSD was stronger among men (odds ratio [OR] = 6.86, 95% CI: 3.11–15.11) than women (OR = 2.77, 95% CI: 1.83–4.21). (Peterlin *et al.*, 2011) The authors suggest a buffering effect of estrogen and oxytocin may contribute to these sex differences.

Several shared pathophysiological mechanisms may contribute to the consistent findings regardless of the order in which each condition occurred. Migraine is known to be strongly heritable, and researchers have identified several genetic links predisposing individuals to both conditions. (Amiri *et al.*, 2022; Gormley *et al.*, 2016; Stam *et al.*, 2010) Individuals with sensitive hormonal or central nervous system responses to stress may also be more likely to develop both conditions, regardless of the order in which they present. (Peterlin *et al.*, 2009) Social factors such as childhood mistreatment may contribute to the development of both conditions (Guidetti *et al.*, 2016, 2009; Peterlin *et al.*, 2011a).

We were unable to account for many of these common causes in our analysis without biospecimens or genetic information. While we do collect data on childhood mistreatment, we did not adjust for childhood mistreatment in our analysis as it may have been the traumatic event which led to PTSD, our outcome of interest. We also did not adjust for comorbid mental illnesses such as anxiety and depression, as PTSD in the absence of these conditions is rare. Our results may therefore reflect common causes of migraine and PTSD.

In addition to these limitations, our measurement of migraine was inconsistent over follow-up. Prior to 2007, participants were asked to self-report migraine diagnosis by a healthcare professional, which has been identified as highly specific in a similar cohort. (Schürks *et al.*, 2009) However, after 2007, participants were asked to self-report migraine headaches during varying time periods, which may explain the high prevalence of migraine in our sample compared to previous studies. While participants are nurses and therefore may be more adept in differentiating between migraine and other headache, this change in wording may have resulted in increased sensitivity and decreased specificity of migraine measurement after 2007, biasing our results in an unpredictable direction. Misclassification of PTSD is unlikely, as our measurement was consistent with DSM-5 criteria.

Some degree of misclassification of condition onset is likely, given gaps in assessment years of migraine and use of year of trauma as a proxy for year of PTSD onset. The gaps in migraine assessment years may have led to inaccurate classification of which condition occurred first for individuals who experienced migraine and trauma close together in time. If migraine onset occurred later than assumed, this would cause our migraine-to-PTSD association to be underestimated and our PTSD-to-migraine association to be overestimated, but would not change our conclusions. In a sensitivity analysis, we set migraine to the earliest possible first occurrence and found a crude OR of 1.67 (95% CI: 1.51–1.84) for the association between migraine and incident PTSD and 1.17 (95% CI: 1.06–1.30) for the association between PTSD and incident migraine. To assess the validity of estimating PTSD onset using age at worst event, we examined data from a previous trauma questionnaire in the NHS II cohort which collected data on year of worst event and year of first PTSD reaction. We found that these events happened in the same year for over 70% of participants.

Results of our sensitivity analyses evaluating trauma and PTSD data reported in 2008 and the risk of new-onset migraine in 2009 or 2013 were largely consistent with our main analysis. While the sensitivity analyses did not show a stronger relationship between PTSD symptoms and migraine with aura, compared to migraine without aura, there were small numbers of incident migraine with aura after 2008, so estimates were imprecise.

## Conclusion

Overall, our findings indicate that PTSD and migraine were strongly comorbid, PTSD is associated with incident migraine, and migraine is associated with incident PTSD. We found that approximately 9% of women with migraine developed PTSD if exposed to trauma, and 49% of individuals with PTSD developed migraines. Future research to identify the role of migraine treatment in the development of PTSD and the role of PTSD treatment in the development of migraine and further explore causal mechanisms for these conditions is warranted, particularly given their high prevalence and burden among women.

## Supporting information

Crowe et al. supplementary material Crowe et al. supplementary material

## Data Availability

Further information including the procedures to obtain and access data from the Nurses’ Health Studies and Health Professionals Follow-up Study is described at (contact email: nhsaccess@channing.harvard.edu) and https://sites.sph.harvard.edu/hpfs/for-collaborators/. Data used in the preparation of this manuscript were submitted to the National Institute of Mental Health (NIMH) Data Archive (NDA). NDA is a collaborative informatics system created by the National Institutes of Health to provide a national resource to support and accelerate research in mental health. Dataset identifier: 10.15154/as7x-0j87. This manuscript reflects the views of the authors and may not reflect the opinions or views of the NIH or of the Submitters submitting original data to NDA.

## Supplementary material

The supplementary material for this article can be found at https://doi.org/10.1017/S2045796024000799.
