# Supplementary material for: Bidirectional analysis of the association between migraine and post-traumatic stress disorder in Nurses’ Health Study II
Source: Epidemiol Psychiatr Sci. 2024 Dec 11;33:e76. doi: 10.1017/S2045796024000799 (PMC11669816; doi:10.1017/S2045796024000799)
Supplement: Crowe et al. supplementary material [file S2045796024000799sup001.docx]

**Supplemental Table 1: Comparison of Study Sample to full NHS2 cohort (2017)**

|  | **Analytic Study Sample** | | **Full NHS 2 Cohort** | |
| --- | --- | --- | --- | --- |
| **Characteristic** | No Reported Migraine  (17,221, 52%) | Ever Reported Migraine  (16,106, 48%) | No Reported Migraine  (37,170, 52%) | Ever Reported Migraine (33,685, 48%) |
| Age in 2017, mean (SD) | 64.8 (4.6) | 64.5 (4.6) | 62.9 (4.6) | 62.6 (4.6) |
| Non-Hispanic white | 96 | 96 | 94 | 95 |
| **Marital Status** | | | | |
| Married, % | 74 | 74 | 74 | 74 |
| Divorced, % | 11 | 12 | 12 | 13 |
| Separated, % | <1 | 1 | <1 | <1 |
| Widowed, % | 6 | 6 | 6 | 6 |
| Partnership, % | 2 | 1 | 2 | 2 |
| Single, % | 6 | 5 | 5 | 4 |
| **Medical/Lifestyle Factors** | | | | |
| Ever Diagnosis of high blood pressure, % | 25 | 28 | 27 | 29 |
| Ever Diagnosis of high cholesterol, % | 28 | 30 | 29 | 32 |
| SSRI^3^ Use % | 12 | 16 | 11 | 15 |
| Alcohol g/day, mean, (SD) | 2.5 (5.6) | 2.1 (4.8) | 3.1 (5.9) | 2.6 (5.4) |
| Current smoker, % | 3 | 3 | 4 | 4 |
| BMI^3^, kg/m², mean (SD) | 27.4 (6.2) | 27.8 (6.3) | 27.4 (6.3) | 27.7 (6.4) |

^1^Self-reported physician-diagnosed migraine (pre-2007), self-reported migraine headache (2007-2020) ^2^Missingness for education level depression was 3%, missingness for marital status, and cigarette smoking was <1%. All other variables had no missing values.

^3^CES-D: Center for Epidemiological Studies Depression Scale. SSRI: Selective Serotonin Reuptake Inhibitor. BMI: Body Mass Index.

**Supplemental Table 2: Migraine Prevalence by Trauma Type**

|  | **Sexual/Interpersonal** | **Accident/Disaster** | **Sudden Death** | **Illness/Injury** | **Nursing** |
| --- | --- | --- | --- | --- | --- |
| Migraine | 7,833 (54%) | 5536 (53%) | 7,536 (50%) | 5,937 (53%) | 4,905 (52%) |
| No Migraine | 6,655 (46%) | 4849 (47%) | 7,436 (50%) | 5,323 (47%) | 4,602 (48%) |
| Total | 14,489 | 10,385 | 14,972 | 11,260 | 9,507 |

**Supplemental Table 3: PTSD Symptoms and incident migraine*- 2008 stress questionnaire**

| **PTSD Symptoms** | **Incident Migraine**  N  (%) | **No Incident Migraine**  N  (%) | **Crude RR**  (95% CI) | **Adj RR***  RR  (95% CI) |
| --- | --- | --- | --- | --- |
| **6-7 PTSD Symptoms** | 100 (7%) | 1,439 | 1.53 (1.23-1.91) | 1.77(1.32-2.37) |
| **4-5 PTSD Symptoms** | 146 (6%) | 2,623 | 1.23 (1.01-1.49) | 1.16 (0.88-1.52) |
| **1-3 PTSD Symptoms** | 204 (5%) | 4,296 | 1.05 (0.88-1.25) | 1.15 (0.92-1.46) |
| **Trauma, no PTSD Symptoms** | 633 (5%) | 13,854 | 1.01 (0.88-1.16) | 1.14 (0.95-1.38) |
| **No trauma** | 278 (5%) | 6,135 | Ref | Ref |

*PTSD symptoms measured in 2008, new onset migraine 2009-2013 **Adjusted for race, marital status, high blood pressure, high cholesterol, alcohol intake, smoking, BMI

**Supplemental Table 4: PTSD Symptoms and incident migraine with aura- 2008 stress questionnaire**

| **PTSD Symptoms** | **Migraine with aura**  **N (%)** | **Migraine without aura**  **N (%)** | **Crude RR**  **(95% CI)** | **Adj RR***  **(95% CI)** |
| --- | --- | --- | --- | --- |
| **6-7 PTSD Symptoms** | 30 (30%) | 70 | 1.37 (0.94-1.98) | 1.28 (0.77-2.14) |
| **4-5 PTSD Symptoms** | 45 (31%) | 101 | 1.49 (1.01-1.95) | 1.66 (1.06-2.58) |
| **1-3 PTSD Symptoms** | 53 (26%) | 151 | 1.18 (0.86-1.63) | 1.36 (0.89- 2.07) |
| **Trauma, no PTSD Symptoms** | 146 (23%) | 487 | 1.05 (0.81-1.37) | 1.06 (0.77-1.53) |
| **No trauma** | 61 (22%) | 217 | Ref | Ref |

*PTSD symptoms measured in 2008, new onset migraine 2009-2013 **Adjusted for race, marital status, high blood pressure, high cholesterol, alcohol intake, smoking, BMI
